# Age-related differences in the relationship between confidence and false memory in a mnemonic discrimination task

**DOI:** 10.1038/s41598-024-82292-z

**Published:** 2025-03-11

**Authors:** Ágnes Szőllősi, Dorottya Bencze, Soma Zsebi, Eszter Juhász, Mihály Racsmány

**Affiliations:** 1 Institute of Psychology, University of Szeged, Szeged, Hungary; 2 Cognitive Medicine Research Group, Competence Centre for Neurocybernetics of the Life Sciences Cluster of the Centre of Excellence for Interdisciplinary Research, Development and Innovation of the University of Szeged, University of Szeged, Szeged, Hungary; 3 Institute of Cognitive Neuroscience and Psychology, HUN-REN Research Centre for Natural Sciences, Budapest, Hungary; 4 Doctoral School of Psychology, Eötvös Loránd University, Budapest, Hungary; 5 Department of Psychology, Eötvös Loránd University, Budapest, Hungary

**Keywords:** False memory, Ageing, Recollection, Mnemonic discrimination, Metamemory, Psychology, Human behaviour

## Abstract

In addition to episodic memory loss there is an increase in false remembering in ageing especially when the discrimination between studied and new items is difficult in a recognition memory task. The aim of this study was to identify the underlying psychological mechanisms of this behavior, specifically, the possible role of false recollection. We used the Mnemonic Similarity Task, a widely used task in neuroscience research developed to assess the behavioral manifestation of hippocampal computations, pattern separation and pattern completion. First, older and young adults (*n* = 39 and 44, respectively) were presented with images of everyday objects. Then, on a surprise recognition test, they saw old (studied) and new (non-studied) items as well as visually similar lures of the images seen in the study phase. Instead of using the original Old/New test format, we asked participants to make confidence judgments. Our response frequency and ROC (receiver operating characteristics) analyses revealed overconfidence in false memories for the lures in the group of older adults suggesting false recollection. Such overconfidence was not observed for the completely new stimuli. Our results imply that older adults tend not to acknowledge some memory problems as a consequence of very high confidence in false memories.

## Introduction

### Episodic memory decline and ageing

It has long been demonstrated that episodic memory declines with age as a consequence of substantial changes in several brain regions, including the prefrontal cortex (see^1^) and the hippocampus (for a recent review, see^2^). Specifically, older adults mainly have difficulties when a task (or situation) requires the conscious recollection of a particular event together with its contextual details. In fact, this could be the reason why in some cases older adults forget someone they have just met or forget that they have already taken their medicines.

**Fig. 1:**
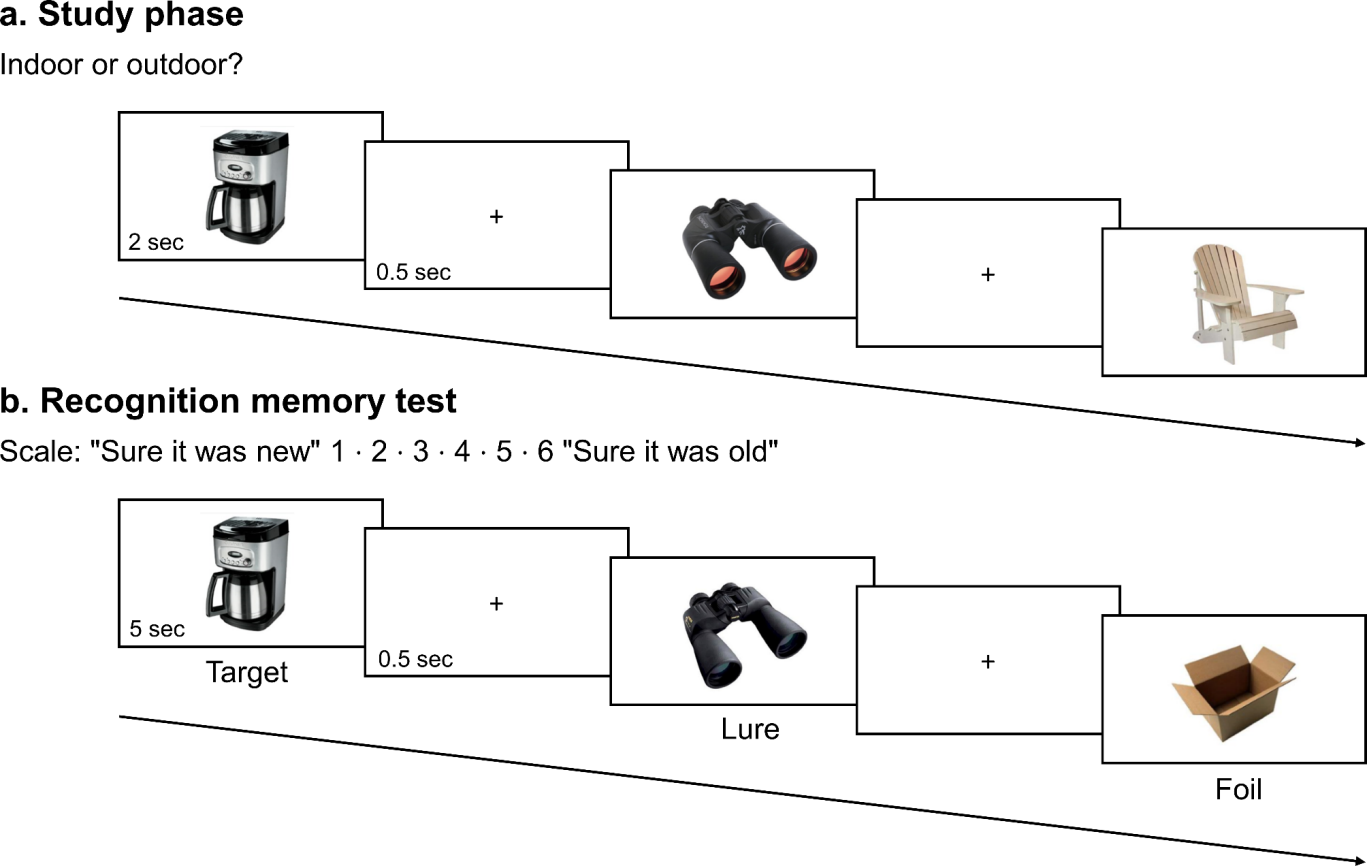
The experimental design and the procedure of the modified Mnemonic Similarity Task. Note(s). Participants were presented with object images in the study phase (**a**), followed by a surprise recognition test where they saw target, lure, and foil images (**b**). The targets were old items, whereas the foils were completely new items. The lures were visually similar to images presented in the study phase. Participants were instructed to make recognition confidence judgments.

Early studies drew attention to an important dissociation with respect to age-related episodic memory problems, demonstrating that older adults mainly have difficulties with retrieval rather than with the encoding of new information^3^. Importantly though, encoding is also affected in aging especially when the encoding of stimulus-specific details is required. In about the last ten years, a modified object recognition memory task, the Mnemonic Similarity Task (MST), became a highly popular test for assessing encoding-related processes in aging^4^. In this task, participants are presented with photographs of everyday objects. This study phase is followed by a recognition test during which participants see old (studied) and new (non-studied) items. Crucially, participants are presented with lure images as well that are visually similar images to ones presented in the study phase. The original studied image and its lure pair can differ in a wide range of characteristics, such as the orientation, size, or color of the object (see Fig. 1 for an example). The correct rejection of a lure stimulus reflects successful discrimination between the studied items and the lures.

The typical pattern of findings in the MST is that older adults have difficulties with lure discrimination whereas have no problem with the correct identification of the studied old items^5–9^. Specifically, older adults tend to identify the lure stimuli as “Old”. This finding suggests that older adults have problems forming unique and detailed memory representations. Instead, they are prone to form general/gist-based memory representations^10^. Successful lure discrimination is suggested to be the behavioral outcome of a computational mechanism of the hippocampus, called pattern separation, whereas the misidentification of a lure stimulus in older adults is proposed to primarily reflect another computational mechanism of the hippocampus, called pattern completion^4,11^. While pattern separation refers to the process by which the neuronal activities of brain circuits become distinct for stimuli that share similar features, pattern completion refers to the activation of stored representations in response to partial or degraded cues^11–14^.

Crucially, individuals often employ the so-called “recall-to-reject” strategy in the MST^15^. That is, when a test stimulus resembles a studied item, individuals try to recall the old item to make a comparison to its lure pair^16,17^. Therefore, pattern completion either can, or cannot, lead to false memory depending on the nature of the representation. On the one hand, pattern completion can lead to correct lure discrimination when one is able to retrieve the original item with its specific details. On the other hand, when the representation is not sufficiently distinct and detailed, the bias towards pattern completion can result in false memory^18^. Several theorists suggest that this shift to pattern completion is responsible for performance change of older adults in the MST, that is, for the increase in the ratio of “Old” responses to the lures^7,9^. In line with results obtained with the MST, several earlier recognition memory studies also showed an increase in false recognition in ageing, especially in cases when the studied old stimuli and the non-studied new stimuli had similar features^19,20^.

### Underlying mechanisms of false recognition in ageing

In the last decades several models have been developed to explain recognition memory and these models can help to better understand the tendency towards false recognition in ageing. The single process models propose that recognition memory decisions are solely based on the level of item familiarity (for overviews, see^21,22^). The dual process models, on the other hand, suggest that in some cases recognition decisions are accompanied by recollection, i.e., by the retrieval of a particular study event together with its contextual details (for overviews, see^23,24^). In some variants of these models^24–26^, recollection is suggested to be a categorical process that occurs when a decision is associated with the highest confidence. In contrast, familiarity-based decisions are typically associated with lower confidence. According to another viewpoint, recollection is not a categorical process. Instead, both recollection and familiarity are continuous, and therefore, some degree of recollection is always present during retrieval^27^. Consequently, high confidence is not solely associated with recollection but with a strong familiarity signal as well. Importantly though, when the recollection signal is strong, retrieval is always associated with high confidence.

Interestingly, the subjective impression of recollection can accompany not only correct but false remembering as well. This phenomenon is often referred to as *false recollection* (for an overview, see^28^). In fact, false recollection is quite frequent when the discrimination between a studied and a new item is difficult^29^. This type of false alarm is typically associated with higher confidence as compared to familiarity-based false recognition^30,31^. This means that sometimes people are prone to strongly believe that they experienced events that never actually happened.

Importantly, false recollection is a key characteristic of age-related memory problems^20,32,33^). A meta-analysis of 27 studies investigating the subjective experience of remembering showed that false recollection is more frequent in older adults as compared to familiarity-based false recognition^34^. More interestingly, this meta-analysis also showed that the ratio of true recollection is comparable to that of false recollection in older adults. Additionally, older individuals usually show high-confidence errors, rather than other age groups^35^. Closely related to this issue, it may be surprising that one can recollect the study context of a new stimulus^31,36^. One possibility for this contradiction could be that false recollection stems from the retrieval of contextual details of a (real) event other than the study event or from recollecting a study event of another, non-presented stimulus^29^.

One way to examine the underlying psychological mechanisms of recognition memory decisions (recollection and familiarity) is the analysis of confidence judgments^22,26^. Importantly, confidence judgments require metamemory, the process of monitoring the progress of memory functioning^37^. Interestingly, despite the close relationship between metamemory and accuracy, they can be separated as indicated by a large number of behavioral studies involving only young adults^38^ and also by a plenty of neuroimaging studies^39^. It has been shown that different processes are involved in true and false recognition associated with high confidence as well as in true and false recognition associated with low confidence. Further evidence for the dissociation of metamemory and accuracy comes from ageing studies. For example, it has been demonstrated that older adults’ false memories are highly similar to true memories in terms of associated sensory and contextual details and that this similarity results in the false sense of recollection as well as source misattributions^40^. In fact, ageing is associated with a decline in monitoring processes that, together with gist-based memories, can lead to false recollection^41^.

### The present study

In brief, in addition to episodic memory loss, there is an increase in false remembering in ageing. In the past few years, the MST became a popular tool to assess this behavior. Typically, older adults show a tendency towards “Old” responses in the MST when they have to mnemonically discriminate between studied items and their visually similar lures. From a behavioral perspective, one possibility is that increased familiarity is responsible for this response bias. Another possibility is that sometimes older adults falsely recollect a related episode instead of the target episode due to their similarities and due to inefficient pattern separation at encoding.

Previous studies have shown that false recollection is more frequent in older adults as compared to familiarity-based false recognition (see e.g.^34^). It has been also shown that false recollection is quite frequent when the discrimination between a studied and a new item is difficult in a recognition memory task (see e.g.^29^), just as in the MST. Therefore, we assumed that false recollection is mainly responsible for older adults’ tendency towards Old responses in the MST. We designed an experiment to test this assumption.

The study of confidence judgments makes it possible to investigate the role of recollection and familiarity in false recognition and also to gain insight into monitoring processes associated with false memory. Therefore, we combined the MST with confidence judgments (see also^6,42,43^) to test the assumption that older adults’ false memories are associated with recollection. Several models of recognition memory propose that high confidence is associated with recollection^26,27^. Therefore, we hypothesized that in the MST unsuccessful lure discrimination would be associated with higher confidence in the older group. Additionally, we assumed that false alarms would be associated with higher confidence in the older group only for the lures and not for the foils.

We asked 100 individuals to participate in the study; finally, 89 individuals volunteered to participate. Following exclusions, the final sample consisted of 39 older adults (aged ≥ 65 years) and a group of 44 young adults (aged ≤ 30 years). We used a modified version of the MST (also used in^43^), see Fig. 1. At first, participants were presented with images of everyday objects and were asked to make Indoor/Outdoor decisions during the study phase of the task. Then, on a surprise recognition memory test, there were studied *target* items, non-studied *foil* items as well as visually similar *lure* images of items shown in the study phase. For each stimulus seen in the study phase, either a target or a corresponding lure image was presented in the test phase. While in the original version of the task participants have two (“Old” and “New”) or three (“Old”, “New”, and “Similar”) response options in the test phase^4^, participants of the present study were asked to make recognition confidence judgments on a scale ranged between “Sure New” (1) and “Sure Old” (6).

## Results

### Data analysis

The datasets generated and analysed during the current study are available in the Open Science Framework (OSF) repository (https://osf.io/ur9qs/?view_only=acc3859d01f34322b4adac0e5473cfd2). To analyze the data, we used the ROC toolbox (the Regents of the University of California, Oakland, California, US) developed by Koen and colleagues^44^ in the MATLAB computing environment (R2018b, the MathWorks, Inc., Natick, Massachusetts, US).

ROC curves (using the dual process signal detection model) were fitted to the individual data. We used the default constrains of the toolbox. We analyzed target-foil discrimination (hits for the targets against false alarms for the foils) and target-lure discrimination (hits for the targets against false alarms for the lures) as well. Area Under the Curve (*AUC*) is suggested to be a good measure of sensitivity due to its multiple advantages^45,46^. Therefore, we used *AUC* to analyze accuracy. *AUC* for target-foil discrimination and *AUC* for target-lure discrimination were calculated for the two groups separately. These values were then analyzed by conducting a 2 × 2 mixed-design analysis of variance (ANOVA) with Discrimination (target-foil and target-lure) as a within-subjects variable and Age (older and young) as a between-subjects variable.

Response frequencies for each confidence level and for the stimulus type of special interest (lures) were also analyzed. A 6 × 2 mixed-design ANOVA was conducted with Confidence level (1, 2, 3, 4, 5, and 6) as a within-subjects factor and Age (older and young) as a between-subjects factor. The aim of this analysis was to gain insight into the underlying mechanisms of recognition decisions for the lure stimuli, since some authors suggest that recollection is associated with the highest confidence, while familiarity is associated with lower confidence^6^.

To examine whether there was an age-related difference only in lure rejections and not in foil rejections, we analyzed response frequencies for the foil items as well. As for the lures, we conducted a 6 × 2 mixed-design ANOVA with Confidence level (1, 2, 3, 4, 5, and 6) as a within-subjects variable and Age (older and young) as a between-subjects variable.

### Discrimination accuracy and response frequencies

**Fig. 2:**
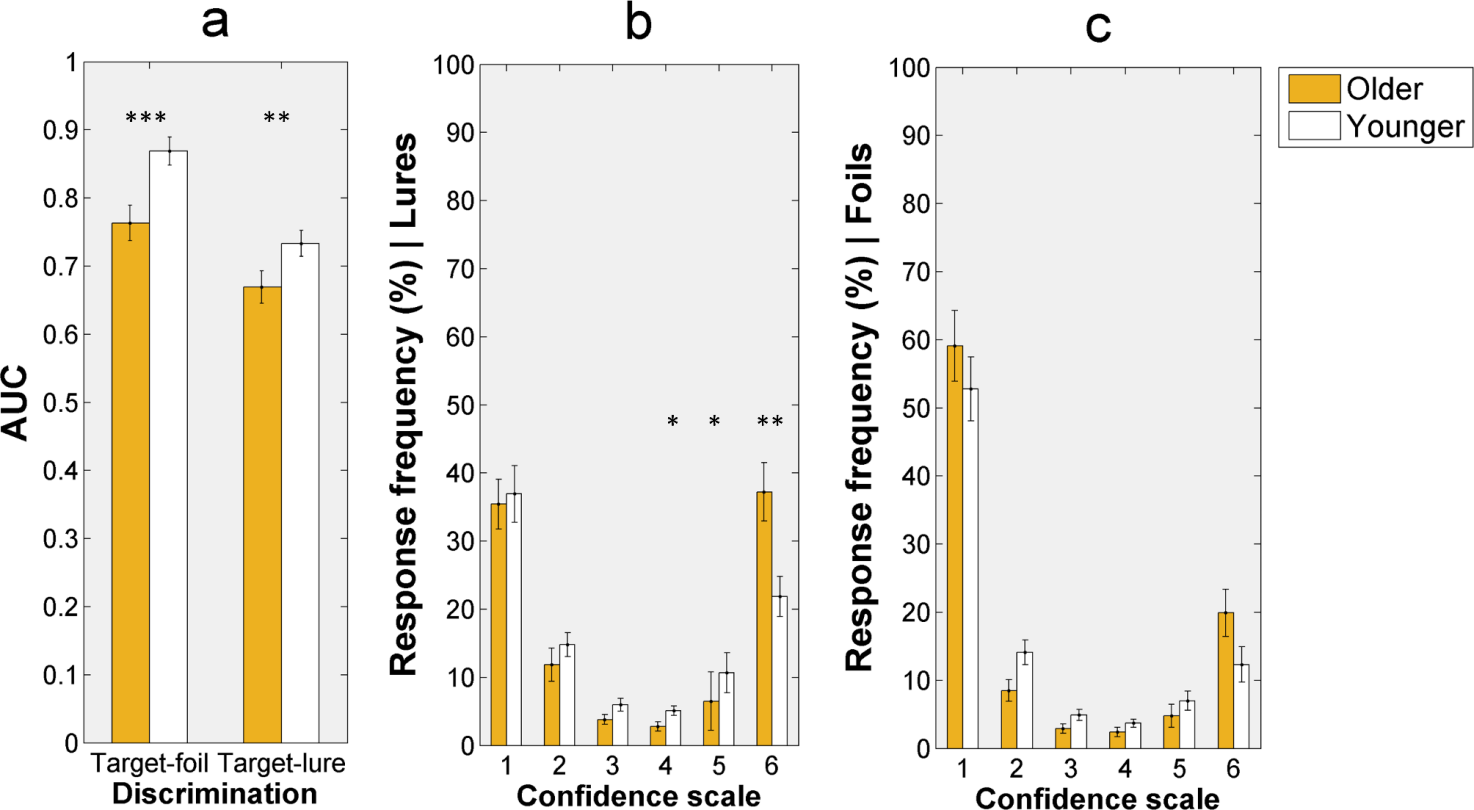
Discrimination accuracy (**a**) and response frequencies for the lures (**b**). Note(s). Targets were the studied old items, foils were the non-studied new items, and the lures were visually similar to images seen in the study phase. AUC = area under the curve. Significant group differences are indicated: *p < .05, **p < .01, ***p < .001. Error bars represent the standard error of the mean.

For discrimination accuracy, as measured by the *AUC*, see Fig. 2a. The ANOVA showed a significant main effect of Discrimination, *F*(1, 81) = 9.608, *p* < .01, η^2^_p_ = 0.106, and a significant main effect of Age, *F*(1, 81) = 52.942, *p* < .001, η^2^_p_ = 0.395. The Discrimination x Age interaction was not significant, *F*(1, 81) = 0.118, *p* = .732, η^2^_p_ = 0.001. This pattern of results indicates that, as expected, target-foil discrimination was better than target-lure discrimination in both groups. In addition, as compared to the older group, young adults were better in discriminating targets from the foils as well as in discriminating targets from the lures. More interestingly, this group difference was comparable for target-foil and target-lure discrimination as indicated by the lack of significant interaction between the independent variables.

Response frequencies to the lure stimuli at each confidence level are seen in Fig. 2b. Confidence, *F*(5, 405) = 43.871, *p* < .001, η^2^_p_ = 0.351, and Age,* F*(1, 81) = 13.609, *p* < .001, η^2^_p_ = 0.144, had main effects on response frequencies. The Confidence x Age interaction was also significant, *F*(5, 405) = 4.667, *p* < .001, η^2^_p_ = 0.054. Post hoc contrast analyses showed that group difference was significant for confidence level 4, 5, and 6 (all *F*s ≥ 4.530, *p*s < .05), and was not significant for confidence level 1, 2, and 3 (all *p*s > 0.05). In brief, older individuals gave false alarms for the lures with the highest confidence level (level 6) more frequently compared to young adults, while in case of lower confidence false alarms (level 4 and level 5) young adults had higher response frequencies.

As expected, Confidence had a significant main effect on response frequencies for the foils, *F*(5, 405) = 102.605, *p* < .001, η^2^_p_ = 0.559, indicating that both groups were able to identify the foil items as new, and these correct responses were more frequent than the incorrect responses. Importantly though, neither the main effect of Age, *F*(1, 81) = 2.475, *p* = .120, η^2^_p_ = 0.030, nor the Confidence level x Age interaction was significant, *F*(1, 81) = 0.098, *p* = .755, η^2^_p_ = 0.001.

## Discussion

The aim of the present study was to investigate the underlying mechanism of mnemonic discrimination and false memory in older adults. For this purpose, we used the MST^4^, a widely used tool developed to assess the behavioral manifestation of hippocampal computations, pattern separation and pattern completion. Instead of using the original test format, we combined the task with recognition confidence judgments.

In accordance with the results of previous studies^6–9^, we found that older participants had difficulties in discriminating between the studied old stimuli and their perceptually similar lures as indicated by the results of our *AUC* analysis. It has been suggested that hippocampal changes are responsible for this widely demonstrated behavioral result (for reviews, see^4,10^). Specifically, as lure discrimination is suggested to be the behavioral manifestation of pattern separation – that supports the formation of unique representations by the hippocampus –, reduced lure discrimination performance is proposed to reflect pattern separation deficit in ageing. Consequently, our findings indicate that the confidence-based variant of the MST is sensitive to age-related decline in hippocampus-dependent memory (see also^6^).

Our frequency analyses revealed age differences in false alarms given to the lures. Specifically, false alarms with the highest confidence were quite frequent in the group of older adults (~ 37%) and were more frequent than it was in the group of young adults (~ 22%). Importantly, we did not find a similar pattern of results for the foils. In other words, age-related differences in correct rejections were present only for novel stimuli with similar features to the studied old items but not for those novel stimuli that were completely new.

The overconfidence in false memories for the lures might imply false recollection^26^. In fact, false recollection can stem from recollecting a study episode of a stimulus other than the test stimulus^29^. Relatedly, it has been suggested that individuals often employ the so-called “recall-to-reject” strategy for lure discrimination in the MST^15^. That is, when a test stimulus resembles a studied item, individuals try to recall the old item to make a comparison to its lure pair^16,17^. Therefore, it seems plausible that older individuals accessed the original stimulus they saw in the study phase of the task and did not notice the difference between the original stimulus and its lure pair. Further corroborating this assumption, older adults tend to form gist-based memory representation with a loss of details^10^ – as a consequence of pattern separation deficits^6^ –, therefore, they may not be able to detect slight perceptual differences between a studied stimulus and a test item. It is important to note that the MST was designed to assess encoding-related processes^4^. Consequently, our findings likely reflect age-related difficulties in encoding. However, since we used only behavioral measures when assessing memory performance, the question of whether our results are solely related to encoding (and not to retrieval-specific processes) remains open. Importantly, young adults also had false alarms, but these responses were mostly associated with lower confidence. In sum, we suggest that our findings indicate that false alarms for the lures were mostly associated with false recollection in the group of older adults and with a false sense of familiarity in the group of young adults.

There are two interrelated factors that contribute to age-related changes in the interplay between confidence judgments and accuracy, as highlighted by Wong and colleagues^41^. First, older adults have difficulty in the retrieval of specific details of previously encountered stimuli. This difficulty can stem from problems with forming detailed representations at encoding^6,7,9^. Second, older adults have impaired metacognitive monitoring processes. These two factors together can lead to false recollection. We suggest that our results corroborate the importance of these two factors in age-related recognition memory problems since we showed that older adults had problems when they had to retrieve specific features of studied stimuli in a discrimination task, and yet, they had a sense of recollection during retrieval attempt. In addition, we suggest that age-related performance change in the MST (i.e., impaired discrimination accuracy) indicates that older adults have problems with accessing the specific features of a given stimulus and that they have limited insight into this problem due to impaired monitoring processes.

We suggest that our results are in line with studies demonstrating that older adults are more likely to make Remember responses for false memories in the Deese-Roediger-McDermott (DRM) paradigm developed to assess semantic false memories^34^. Moreover, these results together with our findings indicate that in older adults recollection accompanies the retrieval of false memories stemming from both perceptual and semantic similarities. Relatedly, a recent study has shown that age-related changes in mnemonic discrimination are not solely based on perceptual factors but are also influenced by semantic similarity^47^.

It should be highlighted that we did not use a control task to compare perceptual discrimination performance between the two age groups. Therefore, the question of whether our results can be explained by perceptual difficulties remains open. However, given that previous studies have demonstrated the MST’s sensitivity to age-dependent changes in mnemonic discrimination, rather than solely perceptual discrimination^9^, we argue that our findings primarily reflect age-related difficulties specific to memory. Future studies are needed to further investigate this question.

Finally, it should be also discussed that, in the present study, older adults had problems with discriminating between the studied and new items as well (as indicated by our *AUC* analysis) despite the lack of considerable perceptual similarity between these stimulus types. Studies using the original two- or three-choice (“Old”/”New” or “Old”/”New”/”Similar”) version of the MST usually found no age effect on the identification of the studied old stimuli^6,7,9^. These former results together with our findings might suggest that confidence ratings are more sensitive to age-related differences in recognition memory performance as compared to the traditional “Old”/”New” (or the “Old”/”New”/”Similar”) test format. Accordingly, several previous studies found age-related recognition memory impairment when examining confidence judgments^35,48^ but not when using the traditional “Old”/”New” test format (for a review, see^3^). One possible explanation for this finding is that confidence decisions require extra monitoring which is known to decline with age^41^. Future studies could investigate this further by comparing the sensitivity of various test formats (confidence judgments vs. Old/New/Similar responses) in older adults.

## General summary and conclusions

False remembering is relatively frequent in situations when one needs to discriminate between similar items. Sometimes, especially in young adults, false memories stem from the false sense of item familiarity. Interestingly though, ageing is associated with a shift to false recollection, as indicated by overconfidence in false memories. Our results imply that older adults tend not to recognize and acknowledge memory problems as a consequence of very high confidence in false memories. It should be an objective of future studies to specify possible solutions to this problem.

## Methods

### Participants

Approximately 100 individuals were invited to participate in the study. Finally, eighty-nine participants volunteered to participate. Required sample size was determined on the basis of previous experiments investigating age differences in mnemonic discrimination using different variants of the MST^4^. One participant was excluded from the sample due to a psychiatric diagnosis and one additional participant was excluded due to a neurological diagnosis. The final sample consisted of individuals with no history of psychiatric and neurological disorders. All participants had normal or corrected-to-normal vision; they did not receive compensation for participation.

Participants were a group of older adults (aged ≥ 65 years) and a group of young adults (aged ≤ 30 years). Older adults completed the Mini-Mental State Examination Test (MMSE^49^) at the time when they volunteered to participate. Most studies suggest to use the cut-off score of 24 or of 25 for the MMSE to minimalize the possibility of involving individuals with cognitive impairment in the sample^50,51^. To further reduce the possibility of involving individuals with cognitive dysfunctions, we used a more strict cut-off score of ≥ 27 points (as suggested by Kukull and colleagues^52^. Due to low MMSE scores, four individuals were excluded from the sample. The MMSE score of the final sample in the older group was as follows: *Mdn* = 29, *IQR* = 2.

Following the exclusions, the final sample consisted of 39 older adults and a group of 44 young adults. The demographic data of the sample are presented in Table 1. There was no statistically significant difference between the groups in the ratio of female/male participants, *χ*^2^ (1, 83) = 0.005, *p* = .942, and in school years, *U* = 742.500, *p* = .187.

**Table 1:** Demographic data of the sample.

		Older adults	Young adults
Gender (females, males)		26, 13	29, 15
Age (in years)	Range	65–82	19–30
	Mean (SD)	69.8 (5.0)	24.5 (2.3)
School years	Range	8–16	8–16
	Mdn (IQR)	12 (0)	12 (4)

All participants gave written informed consent. The study was approved by the Medical Research Council, Hungary. The study was carried out in accordance with the Code of Ethics of the World Medical Association (Declaration of Helsinki) for experiments involving humans.

### The modified Mnemonic Similarity Task: Experimental design and procedure

We used a modified version of the MST (also used in^43^, see Experiment 2). The stimuli were color photographs of everyday objects. The stimulus set was adapted from the database of Stark and colleagues^12^. The task had two phases, an incidental study phase and a recognition memory test, see Fig. 1. Participants were not informed that there would be a memory test.

In the study phase, participants were presented with 128 images, each for 2000 ms (pre-stimulus interval [PSI] = 500 ms). The images were seen in the middle of the computer screen on a white background. Participants were asked to make Indoor/Outdoor decisions by pressing the corresponding response button (F and K, respectively). The response options remained on the screen for the duration of the study phase.

The study phase was followed by a surprise recognition memory test. In this phase, participants were presented with 192 images. Stimulus presentation duration was 5000 ms (PSI = 500 ms). There were three stimulus types: targets, lures, and foils (with 64 images in each condition). 1) The *targets* were exact repetitions of images presented in the study phase; 2) the *lures* were visually similar to images presented in the study phase; 3) the *foils* were completely new images that were not presented at all before. For each stimulus seen in the study phase, either a target or a corresponding lure image was presented in the test phase. Participants were told that they would see not only old and new items, but visually similar pictures to ones seen in the study phase. They were required to make recognition decisions on 6-point confidence scales (where 1 = “Sure it was New”; 6 = “Sure it was Old”) by using the numeric row on a standard computer keyboard. The response options remained on the screen for the duration of the memory test. The memory test was preceded by a 90-s practice phase in which participants saw the labels of the confidence scale (e.g., “Sure it was Old”) and were instructed to press the corresponding response button. Each label remained on the screen for 5000 ms; participants received feedback (“Correct” or “Incorrect” response) following each response in the practice phase.

## Data Availability

The datasets generated and analysed during the current study are available in the Open Science Framework (OSF) repository (https://osf.io/ur9qs/?view_only=acc3859d01f34322b4adac0e5473cfd2).
